# Anlotinib in Chinese patients aged ≥70 years with advanced non-squamous non-small cell lung cancer without prior chemotherapy: a multicenter, single-arm pilot trial

**DOI:** 10.3389/fonc.2024.1335009

**Published:** 2024-04-08

**Authors:** Da Zhao, Zhengguo Li, Xinli Hou, Lei Yang, Zeng Li, Li Yan, Hongling Li, Hua Liu, Xiaoping Liu, Feixue Song, Guixiang Li, Yu Zhang, Xiaoming Hou

**Affiliations:** ^1^Department of Medical Oncology, The First Hospital of Lanzhou University, Lanzhou, China; ^2^Department of Respiratory and Critical Care Medicine, Wuwei Cancer Hospital, Wuwei, China; ^3^Department of Medical Oncology, Hanzhong Central Hospital, Hanzhong, China; ^4^Department of Respiratory Oncology, Gansu Provincial Cancer Hospital, Lanzhou, China; ^5^3201 Hospital Affiliated to Xi’an Jiaotong University School of Medicine, Hanzhong, China; ^6^Department of Oncology, Ankang Hospital of Traditional Chinese Medicine, Ankang, China; ^7^Department of Oncology, Gansu Provincial People’s Hospital, Lanzhou, China; ^8^Department of Respiratory, Gansu Provincial People’s Hospital, Lanzhou, China; ^9^Department of Respiratory, The Second People’s Hospital of Gansu Province, Lanzhou, China; ^10^Department of Medical Oncology, The Second Hospital of Lanzhou University, Lanzhou, China; ^11^Cancer Center, The Second Hospital of Lanzhou University, Lanzhou, China; ^12^Department of Thoracic Surgery, The First Hospital of Lanzhou University, Lanzhou, China

**Keywords:** non-squamous, non-small-cell lung carcinoma, anlotinib, elderly, pilot trial

## Abstract

**Background:**

Based on pharmacoeconomics, drug availability and actual treatment, optimal treatment regimens for Chinese non-small-cell lung carcinoma (NSCLC) patients over 70 years old are needed.

**Methods:**

This multicenter, single-arm pilot trial enrolled patients with advanced non-squamous NSCLC who refused systemic chemotherapy. Eligible patients received anlotinib (12 mg/day, d1-14, Q3W) until disease progression, intolerant toxicities, or withdrawal from the study. The primary endpoint was progression-free survival (PFS).

**Results:**

Forty-nine patients were screened between January 2019 and September 2021, of whom 40 patients were eligible. The median age was 76 years. With a median follow-up period of 16.20 (95% CI: 8.77, 25.10) months, the median PFS was 5.45 months (95% CI: 3.52-9.23) and the median overall survival was 10.32 months (95% CI: 6.44-12.78). Three patients achieved a partial response and 34 had stable disease, with an objective response rate of 7.5% and a disease control rate of 92.5%. Thirty-three (82.5%; 33/40) patients reported treatment-related adverse events (TRAEs) of any grade, and the incidence rate of grade ≥3 TRAEs was 35% (14/40). The most common grade ≥3 TRAEs were hypertension (4/40; 10.0%), hand-foot syndrome (3/40; 7.5%), and proteinuria (2/40; 5.0%).

**Conclusion:**

Anlotinib treatment was feasible and safe in Chinese elderly patients with advanced non-squamous NSCLC who did not receive any systemic chemotherapy.

## 1 Introduction

Non-small-cell lung cancer (NSCLC) is one of the most commonly occurring cancers and the leading cause of cancer death worldwide (1). Its global incidence continues to rise, and the incidence increases with age (2, 3). In 2015, there were 733,300 new lung cancer cases in China and 205,800 cases in the population over 75 years, of which approximately 85% of cases were NSCLC (4). NSCLC patients who were older than 70 years old had greatly reduced life expectancies, with 5-year survival rates as low as 9%-15% (5). Hence, finding optimal regimens for elderly patients with NSCLC is necessary.

Several previous clinical trials explored the efficacy and safety of chemotherapy regimens for elderly NSCLC patients (6–8). However, due to declining organ function, frailty, and other age-related complications, the use of chemotherapy is often limited in elderly patients (9, 10). A previous study found that dual-drug chemotherapy (cisplatin plus gemcitabine or cisplatin plus pemetrexed) improved progression-free survival (PFS) in elderly patients who were >70 years old but increased the treatment-related toxicity (6). Targeted therapy, such as EGFR/ALK tyrosine kinase inhibitor (TKI), might not bring benefit for patients without mutated driven genes. The first approved immune checkpoint inhibitor in China was nivolumab marketing in 2018, nevertheless, the pharmacoeconomics and drug accessibility were poor. In addition, the concerns of immune-related adverse events (AEs) or treatment-related AEs (TRAEs) existed in immunotherapy (11, 12), which might even worsen the prognosis when combined with other regimens in older patients with NSCLC (13). Furthermore, previous clinical trials on targeted therapy or immunotherapy generally recruited patients under 70 years old, and existing evidence with patients >70 years old was mainly retrospective (14–17). Considering the pharmacoeconomics, drug availability and real-world treatment patterns, clinical trials exploring the treatment regimen options for Chinese NSCLC patients over 70 years are warranted.

Anlotinib is a multi-targeting tyrosine kinase inhibitor (TKI) that achieves an anti-tumor effect by targeting vascular endothelial growth factor receptor (VEGFR), platelet-derived growth factor receptors (PDGFR), fibroblast growth factor receptor (FGFR), and C-Kit. Anlotinib was approved in China for the treatment of advanced NSCLC who failed two lines of chemotherapy (18). In a previous phase III randomized clinical trial (ALTER0303), anlotinib improved overall survival (OS) and PFS in patients with NSCLC, including in elderly subpopulations (19). In 2018, National Medical Products Administration (NMPA) officially approved anlotinib monotherapy based on ALTER0303 in patients with advanced NSCLC progressing after second-line or further treatment, providing a new choice for Chinese patients. Recently, some retrospective studies demonstrated the effectiveness and safety of anlotinib in Chinese elderly patients with NSCLC as a first-line therapy setting (20, 21), suggesting that anlotinib could be used as a first-line treatment in Chinese elderly NSCLC patients.

Therefore, considering pharmacoeconomics and drug availability, we hypothesized that anlotinib monotherapy may be a treatment option for Chinese elderly patients with NSCLC. This multicenter, single-arm pilot trial study aimed to elucidate the feasibility of using anlotinib in Chinese elderly patients with advanced non-squamous NSCLC who had not received any prior chemotherapy. The results of this study may provide preliminary evidence for future studies investigating different treatment options in these Chinese patients.

## 2 Methods

### 2.1 Study design and participants

In this multicenter, single-arm pilot trial, elderly patients with advanced non-squamous NSCLC who did not previously receive any systemic chemotherapy were enrolled from Chinese clinical centers between January 2019 and September 2021.

The inclusion criteria were as follows: 1) Patients aged ≥70 years old; 2) Patients with histologically or cytologically confirmed locally advanced or metastatic non-squamous NSCLC (22); 3) Patients who refused chemotherapy but those who received adjuvant chemotherapy and/or neoadjuvant chemotherapy were eligible if the recurrence occurred within six months; 4) Patients without EGFR and ALK alteration, but patients harboring EGFR or ALK mutations who failed prior TKI therapy were eligible; 5) Patients with asymptomatic brain metastases or patients with brain metastases who completed treatment 14 days before the enrollment and had stable disease, but with the confirmation no cerebral hemorrhage by a cranial MRI, CT, or venography; 6) Patients with an ECOG PS score of 0-1.

The exclusion criteria were as follows: 1) Patients with a mixture of small cell lung cancer and NSCLC; 2) Patients with tumors less than 5 mm from a large blood vessel confirmed by CT or MRI, tumors that invaded local blood vessels, or obvious cavitary lung cancers; 3) other conditions deemed unsuitable for inclusion by the investigators, such as those affecting oral medication absorption, presence of severe diseases endangering the security of patients, or conditions influencing the completion of the study.

This trial was conducted according to the principles of the Declaration of Helsinki and Good Clinical Practice guidelines. The study protocol was approved by all ethics committees of participating clinical centers. All patients provided written informed consent before the onset of any procedure. This trial was registered at clinicaltrial.gov (NCT03778853).

### 2.2 Procedures

**Figure 1:**
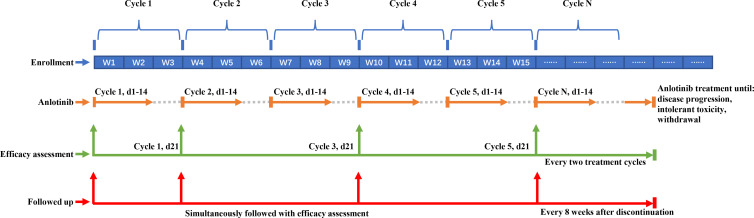
Schematic pepresentation of treatment, efficacy assessment and follow-up. The blue blocks in the top row represent treatment cycles, with every three blocks constituting one cycle (each cycle spans 3 weeks). The corresponding orange lines indicate the administration of anlotinib during the first two weeks of each cycle, continuing until disease progression, occurrence of intolerable toxicity, or withdrawal from the study. Green arrows signify the time points for assessing tumor efficacy. Following the baseline assessment, evaluations are conducted at the completion of the first cycle (day 21), and subsequently every two cycles thereafter. The red line denotes the timeline for follow-up visits. These visits coincide with efficacy assessments during treatment, and continue at 8-week intervals following the discontinuation of treatment.

As shown in **Figure 1**, after enrollment, eligible patients received anlotinib until disease progression, experiencing intolerant toxicity, or withdrawal from the study. Dosage of anlotinib (12 mg/day, d1-14, Q3W) was as previously described (19). For patients with grade ≥3 hematological toxicity or grade ≥2 non-hematological toxicity, anlotinib was interrupted until the toxicity recovered to grade <2 or the dose of anlotinib was adjusted to the lower level (10 mg and 8 mg). The specific dose adjustment scheme referred to the instruction on the use of anlotinib. For patients with progressive disease but evident tumor tissue necrosis or regression and improved or stable cancer-related symptoms, they could continue anlotinib if the investigator deemed that the treatment was beneficial without significant toxicities.

The treatment efficacy was evaluated on the 21st day of the first treatment cycle, and every two treatment cycles thereafter (odd-numbered cycle) according to the Response Evaluation Criteria in Solid Tumors (RECIST) version 1.1 (23). Subjects who discontinued the treatment before disease progression were followed up every 8 weeks and underwent imaging examinations until starting with other anti-cancer treatments.

### 2.3 Endpoints

The primary endpoint was PFS, which was defined as the time from enrollment to the disease progression or death from any cause, whichever occurred first. The secondary endpoints were OS (defined as the time from the enrollment to death from any cause), disease control rate (DCR; defined as the proportion of patients who achieved complete response [CR], partial response [PR], or stable disease [SD] for at least 6 weeks), and the objective response rate (ORR, defined as the proportion of patients who achieved CR or PR to the treatment).

The safety assessment included AE, TRAEs, and serious AE (SAE) that were graded according to Common Terminology Criteria for Adverse Events (CTCAE) version 4.0.

### 2.4 Statistical analysis

Since it was a pilot clinical trial that aimed to investigate the feasibility of using anlotinib for the treatment of elderly patients with advanced non-squamous NSCLC who did not receive prior chemotherapy, the minimum sample size was not calculated.

A full analysis set (FAS) was derived according to the principle of intention-to-treat (ITT), which included all the subjects who took at least one dose of anlotinib. Safety analysis was based on a safety set (SS) that included patients who received at least one dose of anlotinib and had available safety assessment data.

Continuous variables were presented as mean ± standard deviation or median (range). Categorical variables were presented as numbers (percentages). The Kaplan-Meier method was used to estimate the median time and 95% confidence intervals (CIs) for the variables PFS and OS. Statistical analyses were performed using SAS version 9.4.

## 3 Results

### 3.1 Baseline characteristics

**Figure 2:**
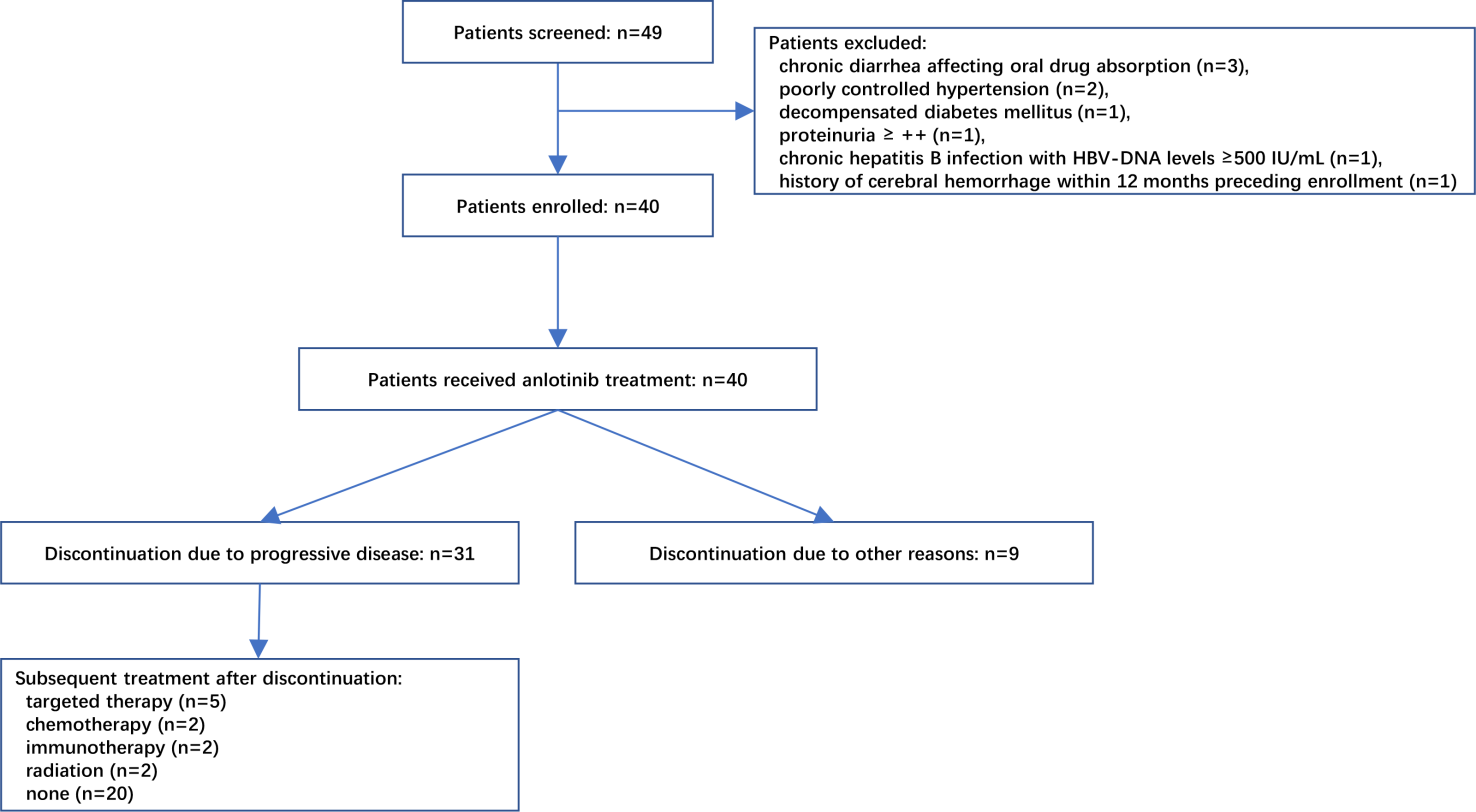
Flowchart of patient recruitment. A total of 49 patients were initially screened, of which 9 were deemed ineligible due to various reasons as illustrated in the diagram. Ultimately, 40 patients were enrolled and received treatment with anlotinib. Among these, 31 patients discontinued treatment due to disease progression, while 11 of them proceeded to receive subsequent targeted therapy, chemotherapy, immunotherapy, or radiotherapy.

As shown in **Figure 2**, 49 patients were assessed for eligibility between January 2019 to September 2021, of whom nine patients were excluded due to various reasons including chronic diarrhea affecting oral drug absorption (n=3), poorly controlled hypertension (n=2), decompensated diabetes mellitus (n=1), proteinuria ≥ ++ (n=1), chronic hepatitis B infection with HBV-DNA levels ≥500 IU/mL (n=1), and a history of cerebral hemorrhage within 12 months preceding enrollment (n=1). Ultimately, 40 patients were enrolled. The median age of patients was 76 years and 52.5% were females. Only five (12.5%) of included patients were with EGFR-mutated NSCLC and experienced failure to previous targeted therapy, and no patient had positive ALK mutation testing. The majority of patients (77.5%) had metastasis. There were also six (15.0%) patients with stage III NSCLC included in this study. These six patients had chosen for oral medication (anlotinib) instead of surgery and radiotherapy, since these treatments were either deemed unsuitable for them or declined by these six patients. The detailed characteristics of patients are presented in **Table 1**.

**Table 1:** Baseline characteristics of patients.

	Total (n=40)
Age, years, median (range)	76 (70-89)
Gender, n (%)	
Male	19 (47.5%)
Female	21 (52.5%)
Cancer stage, n (%)	
III	6 (15.0%)
IV	34 (85.0%)
Smoking history, n (%)	
Yes	20 (50.0%)
No	20 (50.0%)
ECOG-PS, n (%)	
0	11 (27.5%)
1	29 (72.5%)
EGFR mutation, n (%)	
Negative	35 (87.5%)
Positive	5 (12.5%)
ALK mutation, n (%)	
Negative	40 (100.0%)
Positive	0
Metastasis, n (%)	
Yes	31 (77.5%)
No	9 (22.5%)
Metastasis sites, n (%)	
Brain	8 (19.5%)
Bone	11 (26.8%)
Liver	7 (17.1%)

### 3.2 Treatment efficacy

**Figure 3:**
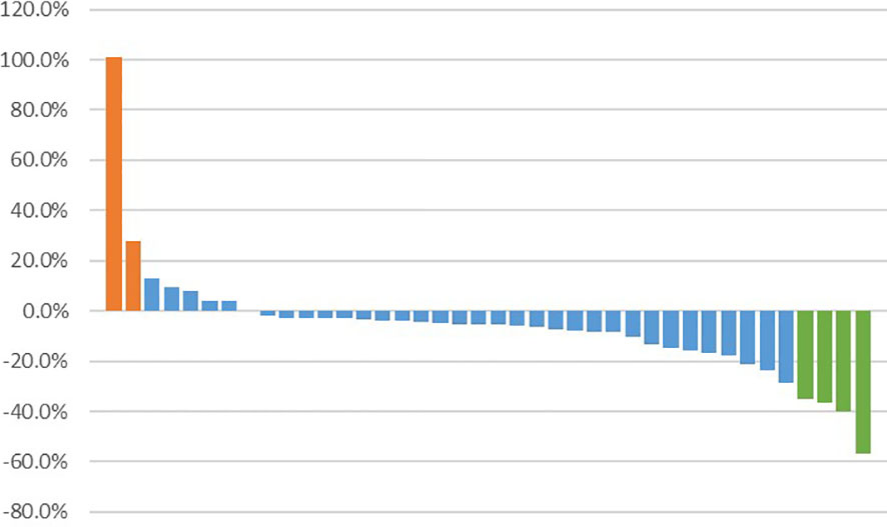
Best percentage change from baseline in target lesion size. Bar representations along the x-axis correspond to individual patients, while the y-axis denotes the extent of tumor size percentage change from baseline to maximum. Patients achieving partial response are depicted in green, those with stable disease in blue, and patients experiencing disease progression in orange.

**Figure 4:**
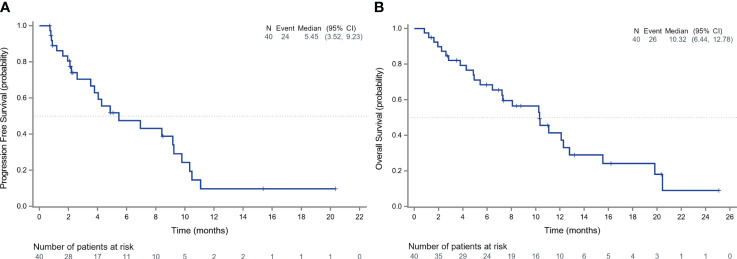
Survival outcomes of patients receiving anlotinib. The Kaplan-Meier curves depict the progression-free survival **(A)** and overall survival **(B)** of all patients receiving anlotinib.

Among 40 patients evaluated for treatment efficacy, three patients achieved PR, with an ORR of 7.5% (3/40). Thirty-four patients were stable with the disease and the DCR was 92.5% (37/40) (**Figure 3**). With a median follow-up of 16.20 months (95%CI: 8.77-25.10), 24 patients were evaluated for PFS events and the median PFS was 5.45 months (95% CI: 3.52-9.23) (**Figure 4A**).

After discontinuing anlotinib, 50% of the patients (20/40) did not receive any other treatments. Five patients received targeted therapy, 2 received chemotherapy, 2 received immunotherapy, and 2 received radiation therapy. During the follow-up, 26 deaths occurred and the median OS in the total population was 10.32 months (95% CI: 6.44-12.78) (**Figure 4B**).

### 3.3 Safety

Thirty-three (82.5%) patients experienced TRAEs of any grade, and the incidence rate of grade ≥3 TRAEs was 35.0% (14/40). As demonstrated in **Table 2**, the most common TRAEs of any grade were hypertension (21/40; 52.5%), followed by hand-foot syndrome (15/40; 37.5%), and fatigue (9/40; 22.5%). The most common TRAEs of grade ≥3 were hypertension (4/40; 10.0%), hand-foot syndrome (3/40; 7.5%), and proteinuria (2/40; 5.0%). The dose was reduced in two cases of proteinuria and one case of hand-foot syndrome.

**Table 2:** Treatment-related adverse events (n=40).

	Any grade, n (%)	Grade ≥3, n (%)
TRAEs	33 (82.5%)	14 (35.0%)
TRAEs ≥5% incidence		
Hypertension	21 (52.5%)	4 (10.0%)
Hand-foot syndrome	15 (37.5%)	3 (7.5%)
Fatigue	9 (22.5%)	0
Proteinuria	7 (17.5%)	2 (5.0%)
Hypothyroidism	5 (12.5%)	1 (2.5%)
Nausea	4 (10.0%)	0
Decreased platelet count	4 (10.0%)	1 (2.5%)
Hypoalbuminemia	3 (7.5%)	0
Hypercholesterolemia	3 (7.5%)	0
Hyperlipidemia	3 (7.5%)	0
Anemia	3 (7.5%)	0

## 4 Discussion

In this pilot clinical trial, Chinese elderly patients with advanced non-squamous NSCLC who did not previously receive chemotherapy were treated with multi-targeting TKI anlotinib. Based on the follow-up data for 16.20 months, anlotinib showed promising anti-tumor effect, with a median PFS of 5.45 months and a median OS of 10.32 months. The safety profile of anlotinib in the elderly population were favorable, with the most common grade ≥3 TRAE of hypertension. To the best of our knowledge, this was the first prospective study of anlotinib to report encouraging safety and efficacy outcomes in Chinese elderly NSCLC patients. Therefore, anlotinib could be considered an alternative oral first-line treatment for Chinese elderly patients with NSCLC who were intolerable to chemotherapy.

Although the immunotherapies showed greater benefit in NSCLC patients without driving gene mutation, previous clinical trials on immunotherapy drugs generally recruited patients under the age of 70 years, and the existing evidence on the elderly population was mainly retrospective (14, 15) and inconsistent (24, 25). As the first commercially available immune checkpoint inhibitor in China, nivolumab was approved for second-line treatment of patients with NSCLC in China in 2018, which opened a new era of NSCLC immunotherapy in China. Around 2018, the pharmacoeconomics and drug availability of immune checkpoint inhibitors in China were poor. Meanwhile, chemotherapy alone was used as first-line treatment in NSCLC (26–28), especially in patients with contraindications to immune checkpoint inhibitors (10). But controversies with chemotherapies still existed in elderly patients with NSCLC due to the co-existing age-related complications and other diseases (29, 30). Chemo-free treatment options are warranted.

Previous clinical trials of elderly patients with NSCLC mainly focused on chemotherapy with or without bevacizumab (6, 27, 28, 31, 32). In this study, the median PFS of 5.45 months of anlotinib was numerically similar to single-agent chemotherapy (3.9-4.1 months) (27), doublet chemotherapy (2.8-4.9 months) (6, 28, 31, 32), and chemotherapy plus bevacizumab (5.9 months) (32). In addition, the median PFS of anlotinib was also numerically similar to 5.4-7.1 months of pembrolizumab monotherapy in the driver-gene wild-type NSCLC with different PD-L1 expressions (24). In addition, survival advantages of pembrolizumab monotherapy over chemotherapy were demonstrated in patients over 65 years (24). Nevertheless, in a meta-analysis, pembrolizumab or atezolizumab monotherapy as first-line or second-line therapy showed no survival benefit for patients aged ≥75 years with NSCLC (25). The present study yields numerically similar survival benefits compared with chemotherapy or immunotherapy, supported by previous literature (21). Noticeably, 50% of the patients in this study didn’t receive any other anti-tumor treatments after the discontinuation of anlotinib, which might have contributed to the underestimation of the survival advantage of anlotinib. Overall, anlotinib might be an effective alternative for Chinese elderly patients with advanced NSCLC, especially for patients unsuitable or intolerant to immunotherapy or chemotherapy.

In exploring the effectiveness of anlotinib as a first-line treatment for NSCLC across different age demographics, a real-world study has discovered that anlotinib, when administered as monotherapy, offers comparable benefits in terms of PFS and OS for both younger patients (under 70 years) and older patients (aged 70 years and above) (33). This finding is particularly significant for elderly patients, who are frequently underrepresented in clinical trials and may encounter unique challenges, including an elevated likelihood of chemotherapy intolerance. It highlights the importance of anlotinib as a treatment option, especially for those who are ineligible for more aggressive therapies. Moreover, a prospective, three-arm study investigating the combination of anlotinib with either EGFR-TKI, chemotherapy, or immune checkpoint inhibitors (ICIs) demonstrated notable tolerability and promising efficacy as first-line therapies in NSCLC patients (34). These findings reinforce the viability of anlotinib-based treatments across a wider patient population. However, given the specific considerations for our elderly cohort, including chemotherapy intolerance and the practical challenges associated with immunotherapy, such as high costs and limited availability, further research is imperative to determine the suitability and tolerability of these combination therapies in elderly.

In the elderly NSCLC cohort, paclitaxel yielded an ORR of 34.6% and a DCR of 80.8% (35), and paclitaxel/carboplatin achieved an ORR of 29.4% and a DCR of 78.0% (36). The checkpoint inhibitors were effective, and they may be appropriate for use in elderly NSCLC patients, especially those with impaired functional status (37). Although the ORR in this study was only 7.5%, the DCR was as high as 92.5%, similar to the previous results obtained for anlotinib in NSCLC (19, 38). Anlotinib is beneficial for Chinese elderly patients, especially those who cannot refuse chemotherapies and those who cannot choose immunotherapy for financial reasons.

Besides the anti-tumor activity, the safety profile is considered crucial when selecting the optimal regimen for elderly patients. In this study, 35.0% of patients had grade ≥3 TRAEs. This incidence was lower than that in elderly patients with chemotherapy alone or in combination with bevacizumab (6, 27, 28, 31, 32), indicating a better safety profile. In this study, the most common TRAEs of any grade were hypertension, hand-foot syndrome, and fatigue. The most common grade ≥3 TRAE was hypertension (10.0%). The AE profile was consistent with previous studies of anlotinib in elderly patients (38, 39) and young patients (19). No new safety signals were identified. A low rate of dose adjustment (7.5%) also indicated the good tolerability of, anlotinib in elderly patients with NSCLC.

This study acknowledges some limitations as well. Firstly, this trial was a pilot study with a rather small number of samples, which might not provide sufficient statistical power. For instance, the number of PR cases (n=3) limited the further exploration on the detailed features of this population that obtained significantly benefit from treatment. Despite this, as a pilot study, it offers valuable insights into anlotinib’s potential as a first-line treatment for Chinese elderly patients with NSCLC. This preliminary evidence supports further exploration of anlotinib’s effectiveness and safety within this new indication setting. Secondly, the absence of testing for PD-L1 and various other driver genes may impact the generalizability of our findings to current clinical settings, where these often guides treatment decisions.

In conclusion, Anlotinib was feasible and safe in Chinese patients over 70 years old with advanced non-squamous NSCLC who did not receive systemic chemotherapy. Therefore, anlotinib might be an effective alternative for Chinese elderly patients with non-squamous NSCLC, especially for those unsuitable or intolerant to immunotherapy or chemotherapy.

## Data availability statement

The original contributions presented in the study are included in the article/supplementary material. Further inquiries can be directed to the corresponding author.

## Ethics statement

The study protocol was approved by all ethics committees of participating clinical centers. The studies were conducted in accordance with the local legislation and institutional requirements. Written informed consent for participation in this study was provided by the participants’ legal guardians/next of kin.

## Author contributions

DZ: Conceptualization, Data curation, Formal analysis, Investigation, Methodology, Validation, Visualization, Writing – original draft, Writing – review & editing. ZL: Data curation, Formal analysis, Investigation, Visualization, Writing – original draft, Writing – review & editing. XLH: Data curation, Investigation, Validation, Writing – original draft, Writing – review & editing. LY: Data curation, Investigation, Writing – original draft, Writing – review & editing. ZL: Data curation, Writing – original draft, Writing – review & editing. YL: Data curation, Investigation, Writing – original draft, Writing – review & editing. HLL: Data curation, Investigation, Writing – original draft, Writing – review & editing. HL: Data curation, Investigation, Writing – original draft, Writing – review & editing. XL: Data curation, Investigation, Writing – original draft, Writing – review & editing. FS: Data curation, Investigation, Writing – original draft, Writing – review & editing. GL: Data curation, Investigation, Writing – original draft, Writing – review & editing. YZ: Data curation, Investigation, Writing – original draft, Writing – review & editing. XMH: Conceptualization, Data curation, Investigation, Validation, Writing – original draft, Writing – review & editing.

## References

[1] Sung H Ferlay J Siegel RL Laversanne M Soerjomataram I Jemal A. Global cancer statistics 2020: GLOBOCAN estimates of incidence and mortality worldwide for 36 cancers in 185 countries. CA Cancer J Clin. (2021) 71:209–49. doi: 10.3322/caac.21660 33538338

[2] Rodak O Peris-Díaz MD Olbromski M Podhorska-Okołów M Dzięgiel P. Current landscape of non-small cell lung cancer: epidemiology, histological classification, targeted therapies, and immunotherapy. Cancers (Basel). (2021) 13(18):4705. doi: 10.3390/cancers13184705 34572931 PMC8470525

[3] Barta JA Powell CA Wisnivesky JP. Global epidemiology of lung cancer. Ann Glob Health. (2019) 85(1):8. doi: 10.5334/aogh.2419 30741509 PMC6724220

[4] Chen W Zheng R Baade PD Zhang S Zeng H Bray F. Cancer statistics in China, 2015. CA Cancer J Clin. (2016) 66:115–32. doi: 10.3322/caac.21338 26808342

[5] Atagi S Mizusawa J Ishikura S Takahashi T Okamoto H Tanaka H. Chemoradiotherapy in elderly patients with non-small-cell lung cancer: long-term follow-up of a randomized trial (JCOG0301). Clin Lung Cancer. (2018) 19:e619–e27. doi: 10.1016/j.cllc.2018.04.018 29887243

[6] Gridelli C Morabito A Cavanna L Luciani A Maione P Bonanno L. Cisplatin-based first-line treatment of elderly patients with advanced non-small-cell lung cancer: joint analysis of MILES-3 and MILES-4 phase III trials. J Clin Oncol. (2018) 36:2585–92. doi: 10.1200/JCO.2017.76.8390 30028656

[7] Okamoto I Nokihara H Nomura S Niho S Sugawara S Horinouchi H. Comparison of carboplatin plus pemetrexed followed by maintenance pemetrexed with docetaxel monotherapy in elderly patients with advanced nonsquamous non-small cell lung cancer: A phase 3 randomized clinical trial. JAMA Oncol. (2020) 6:e196828. doi: 10.1001/jamaoncol.2019.6828 32163097 PMC7068674

[8] Quoix E Audigier-Valette C Lavolé A Molinier O Westeel V Barlesi F. Switch maintenance chemotherapy versus observation after carboplatin and weekly paclitaxel doublet chemotherapy in elderly patients with advanced non-small cell lung cancer: IFCT-1201 MODEL trial. Eur J Cancer (Oxford Engl 1990). (2020) 138:193–201. doi: 10.1016/j.ejca.2020.07.034 32898792

[9] Almodovar T Teixeira E Barroso A Soares M Queiroga HJ Cavaco-Silva J. Elderly patients with advanced NSCLC: The value of geriatric evaluation and the feasibility of CGA alternatives in predicting chemotherapy toxicity. Pulmonology. (2019) 25:40–50. doi: 10.1016/j.pulmoe.2018.07.004 30266308

[10] Losanno T Gridelli C. First-line treatment of metastatic non-small cell lung cancer in the elderly. Curr Oncol Rep. (2021) 23:119. doi: 10.1007/s11912-021-01105-y 34342732

[11] Nosaki K Saka H Hosomi Y Baas P de Castro G Jr. Reck M. Safety and efficacy of pembrolizumab monotherapy in elderly patients with PD-L1-positive advanced non-small-cell lung cancer: Pooled analysis from the KEYNOTE-010, KEYNOTE-024, and KEYNOTE-042 studies. Lung Cancer. (2019) 135:188–95. doi: 10.1016/j.lungcan.2019.07.004 31446994

[12] Morimoto K Yamada T Yokoi T Kijima T Goto Y Nakao A. Clinical impact of pembrolizumab combined with chemotherapy in elderly patients with advanced non-small-cell lung cancer. Lung Cancer. (2021) 161:26–33. doi: 10.1016/j.lungcan.2021.08.015 34500218

[13] Hakozaki T Hosomi Y Shimizu A Kitadai R Mirokuji K Okuma Y. Polypharmacy as a prognostic factor in older patients with advanced non-small-cell lung cancer treated with anti-PD-1/PD-L1 antibody-based immunotherapy. J Cancer Res Clin Oncol. (2020) 146:2659–68. doi: 10.1007/s00432-020-03252-4 PMC11804739 32462298

[14] Galli G De Toma A Pagani F Randon G Trevisan B Prelaj A. Efficacy and safety of immunotherapy in elderly patients with non-small cell lung cancer. Lung Cancer. (2019) 137:38–42. doi: 10.1016/j.lungcan.2019.08.030 31526910

[15] Yamaguchi O Imai H Minemura H Suzuki K Wasamoto S Umeda Y. Efficacy and safety of immune checkpoint inhibitor monotherapy in pretreated elderly patients with non-small cell lung cancer. Cancer Chemother Pharmacol. (2020) 85:761–71. doi: 10.1007/s00280-020-04055-7 32193618

[16] Yamada Y Imai H Sugiyama T Minemura H Kanazawa K Kasai T. Effectiveness and safety of EGFR-TKI rechallenge treatment in elderly patients with advanced non-small-cell lung cancer harboring drug-sensitive EGFR mutations. Medicina (Kaunas). (2021) 57(9):929. doi: 10.3390/medicina57090929 34577852 PMC8466413

[17] Furuta H Uemura T Yoshida T Kobara M Yamaguchi T Watanabe N. Efficacy and safety data of osimertinib in elderly patients with NSCLC who harbor the EGFR T790M mutation after failure of initial EGFR-TKI treatment. Anticancer Res. (2018) 38:5231–7. doi: 10.21873/anticanres.12847 30194172

[18] Zhou M Chen X Zhang H Xia L Tong X Zou L. China National Medical Products Administration approval summary: anlotinib for the treatment of advanced non-small cell lung cancer after two lines of chemotherapy. Cancer Commun (Lond). (2019) 39:36. doi: 10.1186/s40880-019-0383-7 31221221 PMC6585030

[19] Han B Li K Wang Q Zhang L Shi J Wang Z. Effect of anlotinib as a third-line or further treatment on overall survival of patients with advanced non-small cell lung cancer: the ALTER 0303 phase 3 randomized clinical trial. JAMA Oncol. (2018) 4:1569–75. doi: 10.1001/jamaoncol.2018.3039 PMC6248083 30098152

[20] Wang W Shao L Xu Y Song Z Lou G Zhang Y. Efficacy and safety of anlotinib with and without EGFR-TKIs or immunotherapy in the treatment of elder patients with non-small-cell lung cancer: a retrospective study. BMC Pulm Med. (2022) 22:179. doi: 10.1186/s12890-022-01981-5 35524294 PMC9074279

[21] Zhu J Xie Q Zhong A Le Y. Clinical analysis of anlotinib as first-line treatment for elderly patients with advanced lung adenocarcinoma without driver gene mutations. Anticancer Drugs. (2022) 33:e584–e9. doi: 10.1097/CAD.0000000000001186 34387607

[22] Rami-Porta R Asamura H Travis WD Rusch VW. Lung cancer — major changes in the American Joint Committee on Cancer eighth edition cancer staging manual. CA: A Cancer J Clin. (2017) 67:138–55. doi: 10.3322/caac.21390 28140453

[23] Eisenhauer EA Therasse P Bogaerts J Schwartz LH Sargent D Ford R. New response evaluation criteria in solid tumours: revised RECIST guideline (version 1.1). Eur J Cancer. (2009) 45:228–47. doi: 10.1016/j.ejca.2008.10.026 19097774

[24] Mok TSK Wu YL Kudaba I Kowalski DM Cho BC Turna HZ. Pembrolizumab versus chemotherapy for previously untreated, PD-L1-expressing, locally advanced or metastatic non-small-cell lung cancer (KEYNOTE-042): a randomised, open-label, controlled, phase 3 trial. Lancet. (2019) 393:1819–30. doi: 10.1016/S0140-6736(18)32409-7 30955977

[25] Landre T Des Guetz G Chouahnia K Fossey-Diaz V Culine S. Immune checkpoint inhibitors for patients aged >/= 75 years with advanced cancer in first- and second-line settings: A meta-analysis. Drugs Aging. (2020) 37:747–54. doi: 10.1007/s40266-020-00788-5 32681403

[26] Gridelli C. The ELVIS trial: a phase III study of single-agent vinorelbine as first-line treatment in elderly patients with advanced non-small cell lung cancer. Elderly Lung Cancer Vinorelbine Ital Study. Oncologist. (2001) 6 Suppl 1:4–7. doi: 10.1634/theoncologist.6-suppl_1-4 11181997

[27] Gridelli C Perrone F Gallo C Cigolari S Rossi A Piantedosi F. Chemotherapy for elderly patients with advanced non-small-cell lung cancer: the Multicenter Italian Lung Cancer in the Elderly Study (MILES) phase III randomized trial. J Natl Cancer Inst. (2003) 95:362–72. doi: 10.1093/jnci/95.5.362 12618501

[28] Quoix E Zalcman G Oster JP Westeel V Pichon E Lavole A. Carboplatin and weekly paclitaxel doublet chemotherapy compared with monotherapy in elderly patients with advanced non-small-cell lung cancer: IFCT-0501 randomised, phase 3 trial. Lancet. (2011) 378:1079–88. doi: 10.1016/S0140-6736(11)60780-0 21831418

[29] Meoni G Cecere FL Lucherini E Di Costanzo F. Medical treatment of advanced non-small cell lung cancer in elderly patients: a review of the role of chemotherapy and targeted agents. J Geriatr Oncol. (2013) 4:282–90. doi: 10.1016/j.jgo.2013.04.005 24070465

[30] Locher C Pourel N Le Caer H Berard H Auliac JB Monnet I. Impact of a comprehensive geriatric assessment to manage elderly patients with locally advanced non-small-cell lung cancers: An open phase II study using concurrent cisplatin-oral vinorelbine and radiotherapy (GFPC 08-06). Lung Cancer. (2018) 121:25–9. doi: 10.1016/j.lungcan.2018.04.017 29858022

[31] Blanchard EM Moon J Hesketh PJ Kelly K Wozniak AJ Crowley J. Comparison of platinum-based chemotherapy in patients older and younger than 70 years: an analysis of Southwest Oncology Group Trials 9308 and 9509. J Thorac Oncol. (2011) 6:115–20. doi: 10.1097/JTO.0b013e3181fbebfd PMC3075557 21107287

[32] Ramalingam SS Dahlberg SE Langer CJ Gray R Belani CP Brahmer JR. Outcomes for elderly, advanced-stage non small-cell lung cancer patients treated with bevacizumab in combination with carboplatin and paclitaxel: analysis of Eastern Cooperative Oncology Group Trial 4599. J Clin Oncol Off J Am Soc Clin Oncol. (2008) 26:60–5. doi: 10.1200/JCO.2007.13.1144 18165641

[33] Wang F Jin F Cheng B Zhang Y Zhou Q Wang S. The real-world efficacy and safety of anlotinib in advanced non-small cell lung cancer. J Cancer Res Clin Oncol. (2022) 148:1721–35. doi: 10.1007/s00432-021-03752-x PMC8343360 34357411

[34] Chu T Zhang W Zhang B Zhong R Zhang X Gu A. Efficacy and safety of first-line anlotinib-based combinations for advanced non-small cell lung cancer: a three-armed prospective study. Transl Lung Cancer Res. (2022) 11:1394–404. doi: 10.21037/tlcr PMC9359953 35958322

[35] Liu Y Dong Y Zhu H Jing W Guo H Yu J. Nanoparticle albumin-bound paclitaxel in elder patients with advanced squamous non-small-cell lung cancer: A retrospective study. Cancer Med. (2020) 9:1365–73. doi: 10.1002/cam4.2791 PMC7013054 31876976

[36] Soejima K Naoki K Ishioka K Nakamura M Nakatani M Kawada I. A phase II study of biweekly paclitaxel and carboplatin in elderly patients with advanced non-small cell lung cancer. Cancer Chemotherapy Pharmacol. (2015) 75:513–9. doi: 10.1007/s00280-014-2673-8 25563719

[37] Alexa T Antoniu SA Alexa I Ilie A Marinca M Gafton B. Checkpoint inhibitors in NSCLC for the elderly: current challenges and perspectives. Expert Rev Anticancer Ther. (2021) 21:315–23. doi: 10.1080/14737140.2021.1852933 33244997

[38] Jiang HT Li W Zhang B Gong Q Qie HL. Efficacy and safety of anlotinib monotherapy as third-line therapy for elderly patients with non-small cell lung cancer: A real-world exploratory study. Int J Gen Med. (2021) 14:7625–37. doi: 10.2147/IJGM.S334436 PMC8572099 34754233

[39] Song PF Xu N Li Q. Efficacy and safety of anlotinib for elderly patients with previously treated extensive-stage SCLC and the prognostic significance of common adverse reactions. Cancer Manag Res. (2020) 12:11133–43. doi: 10.2147/CMAR.S275624 PMC7646458 33173346

